# Quinoa, *Chenopodium quinoa*, Provides a New Host for Native Herbivores in Northern Europe: Case Studies of the Moth, *Scrobipalpa atriplicella*, and the Tortoise Beetle, *Cassida nebulosa*

**DOI:** 10.1673/031.008.5001

**Published:** 2008-09-09

**Authors:** Lene Sigsgaard, Sven Erik Jacobsen, Jørgen Lindskrog Christiansen

**Affiliations:** ^1^ Department of Ecology, Faculty of Life Sciences, University of Copenhagen, Thorvaldsensvej 40, DK-1871 Frederiksberg C, Denmark; ^2^ Department of Agricultural Sciences, Faculty of Life Sciences, University of Copenhagen, Højbakkegård Allé 13, DK-2630 Taastrup, Denmark

**Keywords:** insect, *Chenopodium* sp., *Chenopodium album*, herbivory, pest

## Abstract

The Andean grain, quinoa, *Chenopodium quinoa* Willd. (Caryophyllales: Amaranthaceae), is gaining increasing attention as a future food and fodder crop in Denmark and other parts of Europe. Prior to 2005, pest problems in the crop were negligible in Denmark, however native insects may become adapted to this new host. Herbivores feeding on the closely related and very common weed in arable crops *Chenopodium album* L. present a special risk. In 2006 there was a heavy attack of *Scrobipalpa atriplicella* (Röslerstamm) (Lepidoptera: Gelechiidae) larvae in the maturing inflorescence of *C. quinoa.* Gelechiidae are the most important pests on *C. quinoa* in the Andean region. In 2007 another herbivore on *C. album*, the tortoise beetle *Cassida nebulosa* L. (Coleoptera: Chrysomelidae), was a serious problem on *C. quinoa* in southern Jutland. This is the first published record of these two pests on *C. quinoa.* The future pest status of *C. quinoa* in northern Europe is discussed.

## Introduction

One of the most important Andean grain crops is quinoa, *Chenopodium quinoa* Willd. (Caryophyllales: Amaranthaceae). The production of *C. quinoa* is linked to its high nutritional value, and recently the market demand both internally and externally has increased rapidly (Jacobsen et al. 2006). *C. quinoa* has been grown on the University of Copenhagen, Faculty of Life Sciences experimental farms in Taastrup for over ten years. The crop has desirable characteristics including tolerance to adverse growing conditions and balanced nutritional qualities (Jacobsen et al. 2003a,b; 2007), and varieties have been selected that are adapted to the cool temperate climate of Denmark. *C. quinoa* is still not grown commercially in Denmark, but experimental trials and plots for propagation of material are being sown at research stations and by farmers around the country.

Until 2005 no major insect pest problems were observed in the crop, however, in 2006 there was a heavy attack of moths in the maturing inflorescence of *C. quinoa* and in 2007 an attack by chrysomelids on the vegetative stage of the crop.

In its area of origin several herbivorous insects feed on and can damage *C. quinoa* during seed germination up through harvest and seed storage in production areas of South America. The most serious of the Andean pests are *Eurysacca quinoae* Povolný and *E. melanocampta* (Meyrick) (Lepidoptera: Gelechiidae) found in Peru and Bolivia that are responsible for yield losses up to 50% or more (Mujica 1993; Rasmussen et al. 2003). Other major insect pests include a group of cutworms (Noctuidae) and phytophagous and pollen-eating beetles including Chrysomelidae and Curculionidae (Rasmussen et al. 2003). Many *C. quinoa* pests are polyphagous, suggesting that a wide range of potential pests exist if *C. quinoa* is implemented as a crop in Europe. Currently reports from Northern Europe include *Cnephasia* sp. (Tortricidae Lepidoptera), *Aphis fabae* L. (Aphididae Homoptera) and *Lygus rugulipennis* Poppius (Miridae Hemiptera) (Gesinski 2000; Jacobsen 1993), and from Southern Europe *Epitrix subcrinita* Le Conte (Chrysomelidae, Coleoptera), and leafhoppers (Cicadellidae, Homoptera) (Rasmussen et al. 2003). This paper is the first published report of *Scrobipalpa atnplicella* (Röslerstamm) (Lepidoptera: Gelechiidae) and *Cassida nebulosa* L. (Coleoptera: Chrysomelidae) on *C. quinoa.*

## Materials and Methods

In late August 2006, panicles of infested *C. quinoa* were collected at Taastrup, located 15 km east of Copenhagen, latitude 55.1°N. At this point in time the whole field was heavily infested. Larvae were feeding in a silken gallery between young leaves, flowers, and seeds that were spun together. The *C. quinoa* being grown was lines from a breeding programme, based on crossings of Danish cultivais KVL- Q52 and KVL-Q37, with Bolivian large seeded Real varieties. Panicles were transferred to three ventilated boxes (base: 30 by 22 cm, height: 12 cm), each holding 5–10 panicles. The bottoms of the boxes were covered with a layer of fine vermiculite. In each box a 9 cm petri dish with filter papers was regularly wetted to provide moisture. Boxes were kept in a climate cabinet providing temperatures of 22 ± 1 °C day and 12 ± 1 °C night (L: D 20:4), simulating Danish summer conditions. Larvae were provided with fresh *C. quinoa* heads for feeding. After two weeks boxes were transferred to a climate cabinet providing 7 ± 1 °C both day and night with L:D of 8:16, simulating winter conditions. By November, pupae were observed, and by early January boxes were returned to temperatures of 22 ± 1 °C day and 12 ± 1 °C night (L: D 20:4). By mid January adults began to emerge. At least 100 adults were collected from the rearing boxes. All were identified as *S. atriplicella* by Dr. Ole Karsholt, The Zoological Museum of Copenhagen.

In June and July 2007, experimental fields of *C. quinoa* in Southern Jutland (the advisory service of “Sønderjysk Landboforening”, Lat: N 55° 9′ “Long: E 9° 13”) were observed to be severely attacked by tortoise beetles. Different larval instars and adults were collected and identified to be *C. nebulosa* (EPPO 2007; Swiêtojañska 2005).

## Results and Discussion

All moth larvae collected in 2006 were identified as being *S. atriplicella* (Karsholt and Nielsen 1998; Karsholt 2004) It is a common species in Denmark, Finland, Sweden, the Netherlands, Belgium and the British Isles (Bland et al. 2002; De Prins 1998; Jansen 1999; Karsholt et al. 1998). Larvae are found on *Chenopodium album* L., *C. ficifolium*, *Atriplex prostrata*, *A. longipes praecox*, *A. littoralis*, *Atriplex* spp. and all kinds of varieties of *Beta vulgaris* subsp. *vulgaris* (De Prins 1998; Jansen 1999; Fritzsche and Keilbach 1994). *S. atriplicella* has also been recorded from Ohio, USA (Metzler and Zebold 1995). In Mexico it is a pest on the cultivated plants of huauzontle (*Chenopodium nuttalliae* [C. *berlandieri* subsp. *nuttalliae*]) and found feeding on *C. album* (Bautista-Martínez et al. 1995).

In The Netherlands the first generation of adults has its peak in the beginning of May and flies until June. A second generation flies from early July with a peak in August (Jansen 1999). Assuming that the collected larvae were offspring of a second generation, this justifies a cold period during rearing. There was no significant difference in the severity of the *S. atriplicella* infestation in the different breeding lines. It is difficult to estimate the potential yield loss, but as no seeds were seen in any of the *C. quinoa* lines from the crossing programme the pest seems to be able to perform a serious attack on C. *quinoa.* In 2007, *C. nebulosa* caused extensive leaf damage in experimental plots of the *C. quinoa* line KVL-Q52 in Southern Jutland. The species feeds on Chenopodiaceae, is commonly found on *C. album*, and has pest status on beet (EPPO 2007). There were no beet fields in the vicinity of the attacked *C. quinoa*, and the preceding crop had been maize, but the pest was observed in *C. album* in neighbouring cereal crops (Susanne Kjær-Hansen, personal communication). In the valleys of southern Peru, the presence of 10 to 15 adults of *Diabrotica* sp. (Chrysomelidae) per plant caused yield losses of more than 20% in the 1998–1999 season (Rasmussen et al. 2003). However, in this case, the crop recovered quite well from the leaf infestation by harvest (Susanne Kjær-Hansen, pers. comm.).

Jacobsen et al. (2003b) hypothesised that naturally occurring herbivorous complexes with some level of oligophagy may constitute a wide range of potential pests if *C. quinoa* is implemented as a crop in regions outside its origin. After more than ten years with *C. quinoa* being grown in Denmark, it appears that native herbivores may now be adapting to, and opportunistically attacking, *C. quinoa*, which represent an abundant and underutilized food source in their habitat.

Both species feed on *C. album* (Williams 1963, Nagasawa and Matsuda 2005) and on beet (EPPO 2007; Fritzsche et al. 1994), but this is the first recorded observation of a serious attack by *S. atriplicella* on *C. quinoa.* Mines have been seen all years, but never a similar attack of larvae as reported here. Similarly, insignificant infestations of *C. nebulosa* were observed in earlier years, but the attack in 2007 was the first serious one. The close relationship between *C. quinoa* and *C. album* and their co-occurrence in the same fields can significantly ease the transition from this weed to *C. quinoa. C. album* is very common at the experimental station in Taastrup, where it is found within and around the *C. quinoa* fields. The association of both pests with beet, which is a widely grown crop, may further raise the risk of transition to *C. quinoa.*

Several factors suggest that *S. atriplicella* has the potential to become a pest: a) the major pests in the countries of origin are also Gelechidae, b) the niche of *S. atriplicella* is very similar to that of its Latin American relatives, c) one of *S. atriplicella's* hosts, *C. album*, is a common weed in agricultural fields, and d) *C. album* belongs to the same genus as *C. quinoa*, and physically *C. quinoa* resembles a large *C. album.*

Evidence from Chrysomelid infestations in Peru (Rasmussen et al. 2003) suggests that *C. nebulosa* will only be an important pest in years of high densities, and that choice of variety can control this pest (Yábar et al. 2002).

Although the commercial importance of *C. quinoa* as a crop in Denmark and in other European countries is currently negligible, its potential is great, and so is the interest for using this high quality product as both food and animal fodder. It is therefore worthwhile to define the potential damaging effect of new pests, in order to look for adequate means of pest management for this new crop. Should *S. atriplicella* and other pests such as *C. nebulosa* become established as a pest on *C. quinoa*, methods of control, including the possible means of biological control, would need further study. Even in *C. quinoa's* countries of origin, many aspects of the biology of its major pests, the Gelechiids, remain poorly understood.
